# Ophthalmic manifestations and vision impairment in Lassa fever survivors

**DOI:** 10.1371/journal.pone.0243766

**Published:** 2020-12-10

**Authors:** Alexa L. Li, Donald Grant, Michael Gbakie, Lansana Kanneh, Ibrahim Mustafa, Nell Bond, Emily Engel, John Schieffelin, Matthew J. Vandy, Steven Yeh, Jessica G. Shantha

**Affiliations:** 1 Section of Vitreoretinal Disease and Surgery, Department of Ophthalmology, Emory Eye Center, Emory University School of Medicine, Atlanta, Georgia, United States of America; 2 Viral Hemorrhagic Fever Program, Kenema Government Hospital, Kenema, Sierra Leone; 3 Ministry of Health and Sanitation, Freetown, Sierra Leone; 4 Department of Microbiology and Immunology, Tulane University School of Medicine, New Orleans, Louisiana, United States of America; 5 Department of Pediatrics, Tulane University School of Medicine, New Orleans, Louisiana, United States of America; 6 Ministry of Health and Sanitation, National Eye Program, Freetown, Sierra Leone; The Scripps Research Institute, UNITED STATES

## Abstract

The purpose of this study was to describe the ocular findings, structural ocular complications, and vision impairment in a cohort of Lassa fever survivors in Kenema, Sierra Leone. A retrospective, uncontrolled, cross-sectional study of 31 Lassa fever survivors (62 eyes) who underwent an ophthalmic evaluation in January 2018 at the Kenema Government Hospital in Kenema, Sierra Leone was performed. Data collection included demographic information, ocular/systemic symptoms, visual acuity (VA), and ophthalmic examination findings. Main outcome measures included anterior and posterior segment ophthalmic manifestations and level of VA impairment in Lassa fever survivors. Anterior segment findings included cataract (18%) and pterygium (2%), while posterior segment manifestations consisted of glaucoma (6%), preretinal hemorrhage (2%), and lattice degeneration (2%). Findings suggestive of prior sequelae of uveitis included chorioretinal scarring (5%), retinal fibrosis (3%), and vitreous opacity (2%). Visual acuity was normal/mildly impaired in 53 eyes (85%), moderately impaired in 6 eyes (10%), and 3 eyes (5%) were considered blind by the World Health Organization (WHO) criteria. Median VA was worse in Lassa fever survivors with ophthalmic disease findings (p<0.0001) for both anterior segment (p<0.0001) and posterior segment disease (p<0.013). Untreated cataract was a significant cause of visual acuity impairment (p<0.0001). Lassa fever survivors in this cohort were found to have cataract and posterior segment findings that potentially represent sequelae of uveitis associated with visual impairment. Future studies are warranted to improve our understanding of the spectrum of ocular disease in this emerging infectious disease of public health consequence.

## Introduction

Lassa fever, an acute viral hemorrhagic fever, is caused by the Lassa virus (LASV), a member of the *Arenaviridae* family. Lassa fever is endemic to the West Africa countries of Benin, Ghana, Liberia, Guinea, Mali, Sierra Leone and Nigeria, affecting 100,000 to 300,000 people annually, and causing approximately 5,000 deaths per year [1]. A recent Lassa fever outbreak in West Africa in 2018 was the largest in history with nearly 1,900 cases reported from January to May 2018 [2]. A Lassa fever outbreak is currently ongoing in Nigeria and has prompted a public health emergency response given that over 1,000 cases have been reported in 2020 [3]. Disease transmission primarily occurs via contact with exposure to urine or feces of infected *Mastomys* species rodents or via human-to-human transmission via infected bodily fluids placing health care workers at-risk [4].

The typical disease course of Lassa fever occurs after an incubation period of up to 21 days, manifesting with fever, myalgia, fatigue, pharyngitis, nausea, and vomiting. Increased vascular permeability can lead to more fatal complications such as pulmonary edema, myocarditis, encephalopathy, hemorrhage, and shock [5]. While the majority of LASV infections are asymptomatic, up to 20% of cases result in severe disease with multi-organ system failure [6]. Chronic sequelae such as sensorineural hearing loss, encephalopathy, and ataxia have been reported in Lassa fever survivors [7].

Given the significant public health risk, clinical severity of disease, and no known effective countermeasures for Lassa fever, the World Health Organization (WHO) has designated Lassa fever as a high priority disease within the WHO Research and Development Blueprint, an approach to the investigation of emerging infectious diseases that represent future global health threats [8]. Other pathogens designated as high priority by the WHO include SARS-CoV-2 (the etiological cause of coronavirus disease 2019), Ebola virus, and Marburg virus, underscoring the impact of understanding the range of organ system dysfunction, including the eye, prior to epidemic or pandemic spread of disease.

Ophthalmic involvement in the acute phase of Lassa fever disease has been described in few series, and acute findings have included conjunctivitis, conjunctival edema, and subconjunctival hemorrhage [5, 6, 9]. Besides these observations, the spectrum of ocular sequelae of Lassa fever survivors and the clinical implications of LASV infection in the eye are currently unknown. The purpose of this study is thus to retrospectively evaluate a series of Lassa fever survivors evaluated in Sierra Leone and to report the ophthalmic manifestations, structural ocular complications, and vision impairment associated with ocular disease.

## Materials and methods

### Study design and population

A retrospective, cross-sectional study was performed on Lassa fever survivors who underwent an ophthalmic examination in January 2018 in Kenema, Sierra Leone. The initial Lassa fever diagnosis at the time of acute illness was made using an antigen capture enzyme-linked immunosorbent assay (ELISA) [10]. The Kenema Lassa Fever Outreach Team utilized a combination of discharge logs and case investigation logs to identify Lassa fever survivors for an ophthalmic examination. The Viral Hemorrhagic Fever Program at the Kenema Government Hospital has extensive experience in the care and investigation of Lassa fever and maintains one of the world’s few Lassa fever isolation wards and also was a strategic Ebola virus disease (EVD) treatment unit during the West African EVD outbreak from 2014–2016. Given this experience, they were able to safely mobilize patients for ophthalmic examinations when this service was available. This study was approved retrospectively by the Emory University Institutional Review Board and the Sierra Leone Ethics and Scientific Review Committee. The study was compliant with HIPAA and adhered to the tenets set forth by the Declaration of Helsinki.

### Data collection

The medical and ophthalmic records of 31 Lassa fever survivors examined in January 2018 at the Kenema Government Hospital in Kenema, Sierra Leone were retrospectively reviewed. Data collection included demographic data (age and gender), past medical history, past ocular history, date of Lassa fever diagnosis, ocular symptoms at the time of acute disease, and ocular symptoms at the time of ophthalmic examination. A complete review of systems was also performed and included documentation of joint pain, body pain, abdominal pain, back pain, hearing loss, tinnitus, headache, palpitations, and itching. Ocular symptoms recorded included the presence of blurry vision, itching, pain, redness, tearing, loss of near vision, and floaters. Main outcome measures consisted of ophthalmic examination findings including Snellen visual acuity, intraocular pressure, slit lamp examination, and dilated fundus examination with indirect ophthalmoscopy recorded on a standardized case report form.

### Personal protective equipment and eye clinic precautions

All patients were afebrile without signs of acute Lassa fever. Thus, ophthalmic examination was performed in the Kenema Government Hospital Eye Clinic with standard and transmission-based precautions as per Centers for Disease Control and Prevention guidance [11]. Specific precautions included gloves during ophthalmic examination, frequent hand washing and alcohol-based sanitation and disinfection of equipment using 70% alcohol wipes between patients. While a Lassa fever outbreak was ongoing in Nigeria in January 2018, no cases of Lassa fever were documented within Sierra Leone during the period of ophthalmic examination for the patients reported herein.

### Statistical analysis

Descriptive data including demographic information, ocular symptoms, systemic symptoms, and ophthalmic findings were summarized. Snellen visual acuity was converted to logarithm of the minimal angle of resolution (logMAR) for statistical analysis. For eyes with hand motions or worse visual acuity, logMAR conversion was performed using previously described methods [12]. Visual impairment was categorized according the World Health Organization’s classification of visual impairment: normal or mild visual impairment (visual acuity of 20/70 or better), moderate visual impairment (>20/70–20/200), severe visual impairment (>20/200-20/400), or blindness (< 20/400) [13].

Mann-Whitney test was used for univariate comparison of median logMAR visual acuity for between group comparisons for individuals with or without a normal eye exam, presence or absence of anterior segment pathology, posterior segment disease (i.e. retina, optic nerve or vitreous abnormality), and presence or absence of cataract. We also evaluated the relationship between patient-reported complaint of blurred vision and ophthalmic variables of interest, which included at least moderate visual acuity impairment and presence of any ophthalmic complication with Fisher’s exact test (Prism, Graph, San Diego, CA; Microsoft Excel, Seattle, WA). P-value < 0.05 was considered statistically significant for all analyses.

## Results

### Study population characteristics

A total of 31 Lassa fever survivors (62 eyes) underwent an ophthalmic examination in January 2018. All patients were from Sierra Leone and 26% of patients were male with a median age of 35 years (interquartile range [IQR] 19–50). The median time from the date of Lassa fever diagnosis to the date of ophthalmic examination was 10.03 years (IQR 6.0–12.0). Five patients (16.1%) reported eye symptoms including pain, redness, and blurry vision during the acute phase of Lassa fever. At the time of ophthalmic examination, Lassa fever survivors reported ocular symptoms including blurry vision (42%), itching (13%), pain (6%), redness (6%), tearing (6%), loss of near vision (3%), and floaters (3%) (Table 1). Review of systems at the time of the evaluation revealed positive responses to a number of systemic ailments including the following: joint pain (26%), hearing loss (16%), headache (16%), body pain (13%), abdominal pain (13%), back pain (10%), tinnitus (3%), palpitations (3%), and itching (3%) (Table 2). Lassa fever survivors residing in the Kenema district traveled for ophthalmic and systemic health evaluation. The majority were from Panguma (25) while others were from Kenema (2) or unreported (3).

**Table 1:** Frequency of ocular symptoms reported by Lassa fever survivors at the time of evaluation.

Ocular Symptoms	Number of Patients (%)
Blurry Vision	13 (42)
Itching	4 (13)
Pain	2 (6)
Redness	2 (6)
Tearing	2 (6)
Loss of Near Vision	1 (3)
Floaters	1 (3)

**Table 2:** Frequency of systemic symptoms reported by Lassa fever survivors.

Systemic Symptoms	Number of Patients (%)
Joint pain	8 (26)
Hearing Loss	5 (16)
Headache	5 (16)
Body pain	4 (13)
Abdominal Pain	4 (13)
Back pain	3 (10)
Tinnitus	1 (3)
Palpitations	1 (3)
Itching	1 (3)

### Ophthalmic findings

**Fig 1:**
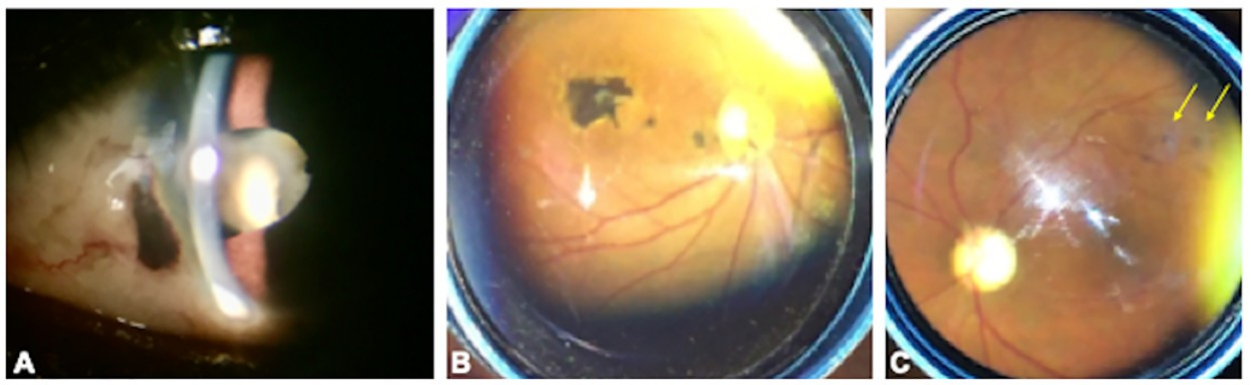
Spectrum of ophthalmic findings in Lassa fever (Lassa fever) survivors. (A) A Lassa fever survivor with a history of a penetrating corneal injury shows uveal prolapse nasally, traumatic cataract and focal posterior synechiae temporally. (B) Another Lassa fever survivor shows macular chorioretinal scar and smaller satellite lesions temporal to the nerve. The visual acuity was 20/200. (C) One Lassa fever survivor shows multifocal scars varying from 100–200 microns within the superotemporal arcade (yellow arrows) but the fovea is spared.

Anterior segment findings included cataract (18%) and pterygium (2%). One patient had a traumatic cataract from penetrating corneal injury, which had self-sealed without surgical repair and showed evidence of uveitis with focal posterior synechiae (Fig 1). This sign of uveitis was deemed to be post traumatic in nature. Besides the patient with a history of trauma, there were no patients who clearly presented with ophthalmic signs consistent with active or inactive anterior uveitis.

Ophthalmic manifestations in the posterior segment included the presence of glaucomatous optic neuropathy (6%), chorioretinal scarring (5%), retinal fibrosis (3%), peripheral drusen (3%), preretinal hemorrhage (2%), lattice degeneration (2%), and vitreous opacity (2%) (Table 3). There were no signs of active intermediate or posterior uveitis at the time of ophthalmic examination in all patients evaluated.

**Table 3:** Ophthalmic findings identified within Lassa fever survivors at the Kenema Government Hospital Eye Clinic.

Ophthalmic Findings	Number of Eyes (%)
Cataract	11 (18)
Glaucoma	4 (6)
Chorioretinal scarring	3 (5)
Retinal Fibrosis	2 (3)
Drusen	2 (3)
Pterygium	1 (2)
Preretinal hemorrhage	1 (2)
Lattice degeneration	1 (2)
Vitreous opacity	1 (2)

### Visual acuity

Visual acuity was normal or mildly impaired in 53 eyes (85%), moderately impaired in 6 eyes (10%), and 3 eyes (5%) were considered blind according to the World Health Organization classification of visual impairment (Table 4). The three eyes that were in the blindness category had hand motion visual acuities attributed to a dense, mature cataract in 1 eye and end-stage glaucoma in 2 eyes. The median logMAR visual acuity of the Lassa fever survivor cohort was 0.00 (Snellen visual acuity equivalent 20/20; IQR 0.00–0.00).

**Table 4:** Visual acuity impairment observed in Lassa fever survivors.

Visual Impairment Category	Number of Eyes (% of total eyes)
Normal/mild visual impairment (better than or equal to 20/70)	53 (85)
Moderate visual impairment (>20/70-20/200)	6 (10)
Severe visual impairment (>20/200-20/400)	0 (0)
Blindness (worse than 20/400)	3 (5)

### Relationship between visual acuity and ophthalmic complications

**Fig 2:**
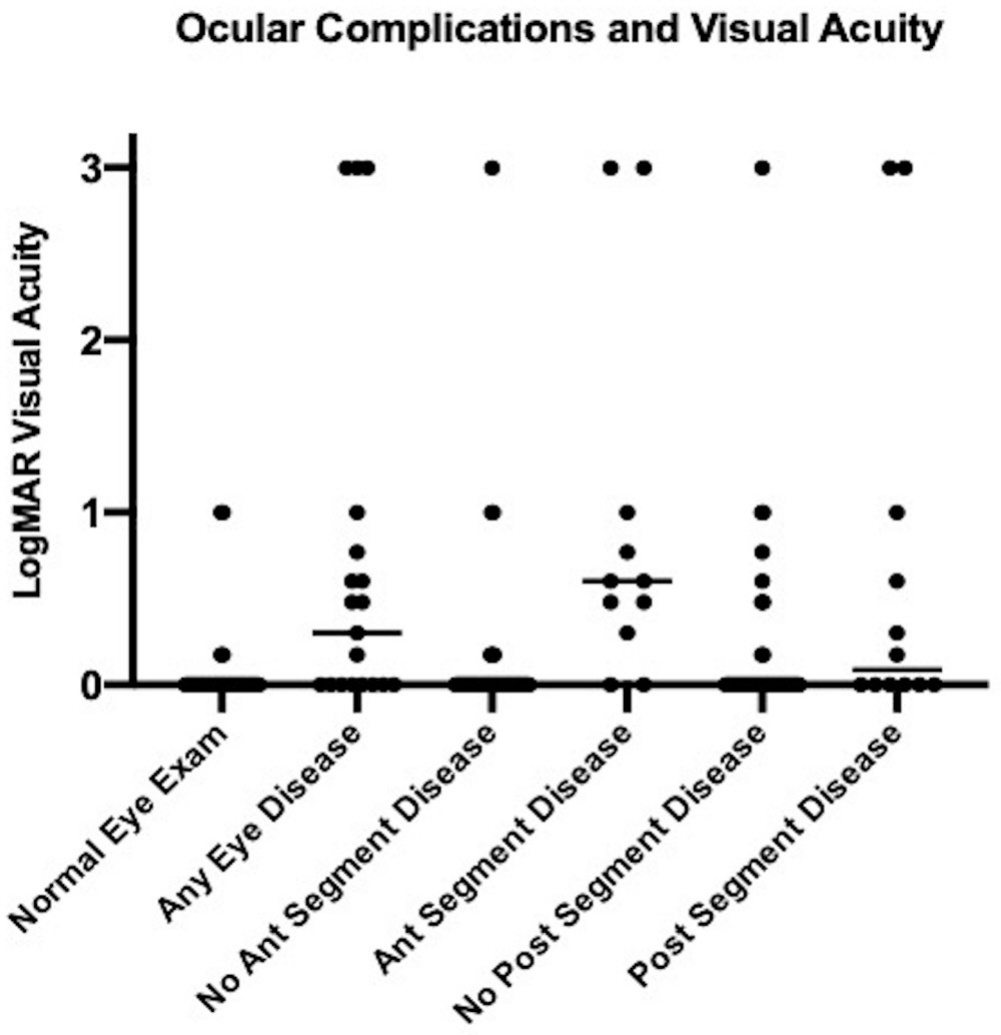
Scatter plot of logMAR visual acuity with medians represented by horizontal bars. Lassa fever survivors with any eye disease showed worse visual acuity than those patients with normal eye examination (p<0.0001). Eyes with anterior segment or posterior segment also showed worse visual acuity when compared to those without disease (p<0.0001 for anterior segment comparison and p<0.013 for posterior segment disease).

We analyzed the impact of ocular structural complications on visual acuity, which are summarized in Table 5 and Fig 2. The median logMAR visual acuity of eyes with any structural complication (0.301, IQR 0–0.769) was approximately 3 lines worse than eyes in which no abnormality was identified (0.0, IQR 0–0, P<0.0001). Eye with anterior segment or posterior segment pathology showed poorer visual acuity than eyes without evidence of anterior (P<0.0001) or posterior segment pathology (P<0.013). Untreated cataract was also a significant cause of visual acuity impairment with worse visual acuity in cataractous eyes (P<0.0001).

**Table 5:** Relationship between visual acuity and ophthalmic disease by anatomic location and impact of untreated cataract.

Comparison Group	Median LogMAR VA (IQR)	P-value
Ophthalmic examination	0.0	
Normal	1.0 (0.00–0.00)	0.0001
Abnormal (any finding)	0.301 (0.00–0.769)	
Anterior segment disease	0.0	
Absent	1.0 (0.00–0.00)	0.0001
Present	0.301 (0.00–0.602)	
Posterior segment disease	0.0	
Absent	1.0 (0.00–0.00)	0.013
Present	0.087 (0.00–0.901)	
Cataract	0.0	
Absent	1.0 (0.00–0.00)	0.0001
Present	0.0 (0.301–0.602)	

IQR–interquartile range; LogMAR–logarithm of the minimum angle of resolution.

### Patient reported visual symptoms, ocular complications, and systemic Lassa fever survivor sequelae

A significant minority of Lassa fever survivors (42%) reported blurred vision and we thus assessed the relationship of this patient-reported symptom to visual acuity impairment and ophthalmic findings. No association was observed between individuals with reported blurred vision and at least moderate visual acuity impairment (i.e. worse than 20/70 acuity, p > 0.05, Fisher’s exact test). In addition, no association was observed between blurred vision and evidence of *any* ophthalmic abnormality by exam (p > 0.05). Individuals with any ocular abnormalities were likely to be older (Median, Interquartile range [IQR]– 50, 37–57 years) compared to patients evaluated without ocular findings (Median age 25 years, IQR 16–38, p<0.0013). Patients who were older tended to report blurred vision symptoms (Median age 45 years, IQR 34–54) than younger patients (Median age 35 years, IQR 18–38, p<0.03). Gender was not associated with the presence of ocular complications or blurred vision symptoms (p>0.05 for both analyses).

Audiovestibular symptoms and joint pain have been reported in Lassa fever survivors and their relationship to ophthalmic disease was analyzed. Seven patients were found to have audiovestibular symptoms (23%) including 6 with hearing loss and 1 patient with tinnitus, which developed after acute Lassa fever. Hearing loss included bilateral disease (1), right ear (2), left ear (1) and unspecified (2). Hearing loss was not associated with patient-reported blurred vision or the presence of posterior segment or anterior segment disease findings (p > 0.05 for all analyses). Joint pain was similarly unassociated with ocular findings (p > 0.05) in our series of patients, although like hearing loss, was observed in a modest proportion (26%) of patients evaluated.

## Discussion

The ophthalmic sequelae of Lassa fever have not been well-characterized, and the aim of this study was to provide a preliminary investigation of the ophthalmic manifestations in Lassa fever survivors. Ocular involvement during the acute phase of Lassa fever has been reported to manifest as conjunctivitis, typically starting on day 7 of illness and lasting for a duration of 5 days [5, 14]. Subconjunctival hemorrhage has also been described in the acute phase of disease and may portend a poorer prognosis [6]. In a case-control study of 441 patients with Lassa fever in Sierra Leone, McCormick et al reported two cases of uveitis that resulted in transient blindness. Interestingly, in one patient, orchitis also developed which may imply active infection in an immune privileged site [15]. Furthermore, in this case-control study, 39.2% of acute Lassa fever patients were diagnosed with conjunctivitis compared to 15.6% of the control cases. In contrast, in our study in which ophthalmic examination was conducted years after acute Lassa fever infection, no cases of conjunctivitis were observed. However, retrospective reporting of symptoms during acute Lassa fever infection revealed that five patients (16.1%) reported eye symptoms including pain, redness, and blurry vision, possibly suggestive of symptoms of acute viral conjunctivitis.

Blurry vision was a common visual complaint with over 40% of patients describing this symptom at the time of ophthalmic evaluation. The majority of patients (85%) had normal or mild vision impairment according to the WHO classification of visual impairment. While McCormick et al identified cases of uveitis with transient vision loss, we did not observe signs of active uveitis in our cohort of Lassa fever survivors who were evaluated [15]. Moreover, while none of the patients showed signs of prior anterior uveitis, there were signs that represented potential sequelae of intermediate and posterior uveitis, which included vitreous opacities (2%), chorioretinal scarring (5%), and retinal fibrosis (3%). One of 3 patients who presented with chorioretinal scarring met WHO criteria for moderate visual impairment with a Snellen visual acuity of 20/200. It is notable that Lassa fever survivors with ophthalmic disease including both anterior and posterior segment disease demonstrated worse visual acuity than individuals who did not manifest these respective disease findings. Their relatedness to Lassa fever requires further characterization.

There are few reports in the literature documenting the prevalence and causes of visual impairment in the general population in West Africa. The Tema Eye Survey, a population-based cross-sectional study of 5,603 participants residing in an urban West African location, reported a 17.1% prevalence of visual impairment and 1.2% rate of blindness in Tema, Ghana, West Africa [16]. Refractive error accounted for the majority (60%) of the visual impairment and blindness in this population, while non-refractive causes of visual impairment included cataract (53.4%), glaucoma (14%), corneal opacification (7.5%), and retinal disease (7.0%). In comparison, in our study, ophthalmic findings within Lassa Fever survivors included cataract (18%), retinal disease (13%), and glaucoma (6%). The prevalence of cataract and glaucoma may have been higher in the Tema Eye Survey due to the older patient population (participants greater than or equal to 40 years of age). It is unknown whether the higher prevalence of retinal disease in our series is related to history of Lassa fever infection. Another hospital-based retrospective study in North Central Nigeria found that the prevalence of allergic conjunctivitis was 38.4% in subjects age 1–16 years and 4.9% in those age greater than 50 years, with the most prevalent comorbid conditions including refractive error (15.4%), pterygium/pinguecula (3.6%), bacterial conjunctivitis (2.2%), glaucoma (2.1%), and cataract (1.3%) [17]. There were no cases of conjunctivitis observed in our cohort of Lassa fever survivors.

Other untreated ophthalmic conditions that were observed in our study of Lassa fever survivors included open globe trauma, cataract and advanced glaucoma. Specifically, cataract and end-stage glaucoma were identifiable causes of blindness (vision loss worse than 20/400) in three patients (9.7%). A dense mature cataract precluded view to the fundus in one of these patients, and thus it is unknown whether there were other posterior segment findings suggestive of cataract formation related to intraocular inflammation or if the cataract was merely age-related. The mechanism of glaucoma is unknown in the two patients with end-stage glaucoma and hand motions visual acuity; however, they did not have visible sequelae of prior ocular inflammation. In comparison, a retrospective study of a hospital population in Sierra Leone, West Africa conducted in 1989 and 1992 revealed that senile cataract was the major cause of blindness (visual acuity less than 3/60) followed by uveitis despite a significant decrease in blindness from onchocerciasis [18]. Blindness from uveitis from causes other than onchocerciasis increased from 6% (14/240) in 1981 compared to 37% (20/54) in 1992. In our study, there were no cases of blindness from uveitis; however, one patient with chorioretinal scarring (a likely sequelae of uveitis) presented with moderate visual impairment.

Long-term systemic symptoms were also present at the time of ophthalmologic examination, including joint pain (26%), hearing loss (16%), and headache (16%). Hearing loss is a well-documented and potentially profound sequelae of Lassa fever, and a literature review of five hospital-based and population-based studies of Lassa fever survivors revealed that approximately one-third (4%-75%) of Lassa virus-infected patients developed sudden sensorineural hearing loss [19]. Our limited analyses did not find a relationship between ocular symptoms and disease findings. The exact mechanism of Lassa virus-mediated hearing loss is unclear, but studies including a murine animal model have reported possible etiologies including direct viral damage during the late acute phase of disease and an immune-mediated injury during convalescence [19, 20].

Animal studies have been utilized to better understand the pathogenesis of LASV in the eye and to investigate the possibility of LASV persistence in this immune-privileged site [9, 21]. Walker et al described LASV recovered from the aqueous humor in the eyes of rhesus monkeys experimentally infected with LASV and reported eyes containing perivascular infiltrates of plasma cells and lymphocytes in the choroid, sclera, iris, and ciliary bodies [21]. Gary et al similarly identified viral nucleic acids by polymerase chain reaction (PCR) in the eye and detected LASV antigen within the anterior uvea in guinea pigs that died of LASV infection through immunohistochemical (IHC) assays [9]. However, whether LASV may persist in the eye is currently unknown.

The few reports of ocular involvement in other members of the *Arenaviridae* family include accounts of similar descriptions of chorioretinal scarring in lymphocytic choriomeningitis virus (LCMV) [22–24]. This hemorrhagic fever virus was first discovered in 1933 and is associated with rodent-transmitted diseases similar to other Arenaviruses [22]. In particular, LCMV can cause congenital infection and has been linked to a cause of chorioretinitis, congenital hydrocephalus, and macrocephaly or microcephaly in children [23]. In a review of 26 serologically confirmed cases of congenital LCMV disease, Wright and colleagues described 21 infants (88%) with chorioretinopathy [23]. Mets et al reported 6 new cases of LCMV congenital chorioretinitis in the United States diagnosed with elevated LCMV antibody titers and normal titers of Toxoplasma gondii, rubella virus, cytomegalovirus, and herpes virus [22]. LCMV infection has also been associated with isolated macular chorioretinal scars mimicking ocular toxoplasmosis in a case report of two children with normal neurological examinations, suggesting that intraocular infection may be the only manifestation of LCMV infection [24]. LCMV-induced chorioretinitis and keratitis in a murine model suggests that the underlying immunopathological mechanism may occur via T cell activation to persistent viral antigens in the eye [25].

Ocular manifestations in viral hemorrhagic fever disease is not limited to Lassa fever and has been described previously with other hemorrhagic fevers including Ebola virus disease (EVD) and Marburg virus disease [26]. A single-center, retrospective cross-sectional study of 96 Ebola Virus Disease (EVD) survivors in Monrovia, Liberia revealed a 22% prevalence of uveitis, with a greater proportion of patients presenting with posterior uveitis and panuveitis specifically [27]. In addition, 10% of patients were diagnosed with cataracts, and 3% were found to have an EVD-associated optic neuropathy [27]. Furthermore, the persistence of Ebola virus in ocular fluid has been observed during convalescence [28, 29], leading to significant clinical, surgical and public health implications [30, 31].

Limitations of our study include the cross-sectional, retrospective nature of the study. Because ophthalmic examination occurred at a single time point, the patient’s baseline eye disease was unknown, and we were unable to ascertain whether the patients’ cataract or chorioretinal scarring occurred as an immediate consequence of Lassa virus infection. Understanding the timing of the ocular findings following acute Lassa virus infection will allow clinicians and investigators to understand the potential relationship between the ocular findings identified and Lassa fever. It is currently unknown whether the etiology of chorioretinal scarring was from prior trauma or other infectious agents such as *Toxoplasma gondii*. Selection bias was also a potential limitation, as patients may have presented for an ophthalmic examination primarily if they reported ocular symptoms. Future controlled, longitudinal studies with larger sample sizes are planned to characterize the prevalence of ocular disease and the spectrum of ophthalmic complications associated with Lassa fever.

In summary, this cross-sectional, retrospective study highlights the first detailed characterization of ophthalmic manifestations in Lassa fever survivors. Evidence of cataract and prior sequelae of intermediate and posterior uveitis were present in this cohort of Lassa fever survivors. Moderate vision impairment was present in 10% of the patients in our study, highlighting the need for further investigation into ophthalmic complications of LASV infection during the acute phase of disease and convalescence. Future studies are warranted to improve our understanding of long-term ophthalmic sequelae and the spectrum of ocular disease in Lassa fever survivors, particularly given its high annual incidence in West Africa and our ophthalmology field’s mandate to understand emerging infectious diseases of public and global health consequence.

## Supporting information

S1 Dataset (XLSX)
